# Methylene blue for vasodilatory shock in the intensive care unit: a retrospective, observational study

**DOI:** 10.1186/s12871-022-01739-w

**Published:** 2022-06-27

**Authors:** Emily E. Naoum, Adam A. Dalia, Russel J. Roberts, Lauren T. Devine, Jamel Ortoleva

**Affiliations:** 1 Department of Anesthesia, Critical Care and Pain Medicine, Massachusetts General Hospital, 55 Fruit Street, GRB 444, Boston, MA 02114 USA; 2 Department of Pharmacy, Massachusetts General Hospital, 55 Fruit Street, Boston, MA 02114 USA; 3 UWorld LLC, 9111 Cypress Waters Blvd, Suite 300, Dallas, TX 75019 USA; 4 Department of Anesthesiology and Perioperative Medicine, Tufts Medical Center, 800 Washington Street Ziskind, Building, 6th Floor, Boston, MA 02111 USA

**Keywords:** Methylene blue, Nitric oxide synthase inhibitors, Vasoplegia, Vasodilatory shock, Vasopressor sparing

## Abstract

**Background:**

Refractory vasodilatory shock is a state of uncontrolled vasodilation associated with underlying inflammation and endothelial dysregulation. Rescue therapy for vasoplegia refractory to catecholamines includes methylene blue (MB) which restores vascular tone. We hypothesized that (1) at least 40% of critically ill patients would respond positively to MB administration and (2) that those who responded to MB would have a survival benefit.

**Methods:**

This study was a retrospective review that included all adult patients admitted to an intensive care unit treated with MB for the indication of refractory vasodilatory shock. Responders to MB were identified as those with a ≥ 10% increase in mean arterial pressure (MAP) within the first 1-2 hours after administration. We examined the association of mortality to the groups of responders versus non-responders to MB. A subgroup analysis in patients undergoing continuous renal replacement therapy (CRRT) was also performed.

Statistical calculations were performed in Microsoft Excel® (Redmond, WA, USA). Where appropriate, the comparison of averages and standard deviations of demographics, dosing, MAP, and reductions in vasopressor dosing were performed via Chi squared, Fisher's exact test, or two-tailed t-test with a p-value < 0.05 being considered as statistically significant. After using the F-test to assess for differences in variance, the proper two tailed t-test was used to compare SOFA scores among responders versus non-responders.

**Results:**

A total of 223 patients were included in the responder analysis; 88 (39.5%) had a ≥ 10% increase in MAP post-MB administration that was not associated with a significant change in norepinephrine requirements between responders versus non-responders (*p*=0.41). There was a non-statistically significant trend (21.6% vs 14.8%, *p*=0.19) toward improved survival to hospital discharge in the MB responder group compared to the non-responder group. In 70 patients undergoing CRRT, there were 33 responders who were more likely to survive than those who were not (*p* = 0.0111).

**Conclusions:**

In patients with refractory shock receiving MB, there is a non-statistically significant trend toward improved outcomes in responders based on a MAP increase >10%. Patients supported with CRRT who were identified as responders had decreased ICU mortality compared to non-responders.

## Background

Vasodilatory shock is an important problem in the intensive care unit that is associated with multiple coexisting disease pathologies. Refractory vasodilatory shock, or vasoplegia, is a state of uncontrolled vasodilation thought to be chiefly mediated by dysregulation of nitric oxide (NO) and soluble guanylate cyclase (sGC) and likely also associated with underlying inflammation and endothelial dysregulation [1, 2]. NO is generated via nitric oxide synthase and results in vasodilation due to the production of cyclic guanosine monophosphate production (cGMP) which subsequently mediates vascular smooth muscle relaxation [1, 3]. Definitions of vasoplegia vary, but most sources define it as requiring vasopressor doses ranging from above 0.2 mcg/kg/min to 0.5 mcg/kg/min of norepinephrine equivalents or more, with a cardiac index (CI) of at least 2.2 L/min/m^2^ and difficulty maintaining a mean arterial pressure (MAP) above 65 mmHg [2, 4, 5]. The use of CI to define vasoplegia can make diagnosis difficult given the trend in less frequent use of pulmonary artery catheters.

Rescue therapy for vasoplegia refractory to catecholamines includes vasopressin, methylene blue (MB), corticosteroids, angiotensin II, ascorbic acid, and hydroxocobalamin [1, 3, 5]. The routine use of these adjunct agents has not been standardized due to limited, inconsistent data and cost. Methylene blue (ProvayBlue™) inhibits endothelial nitric oxide synthase (eNOS), inducible nitric oxide synthase (iNOS), sGC, and cytokines such as tumor necrosis factor-α (TNF-α) [1, 6, 7]. MB restores vascular tone and due to the selective blockade of both sGC and iNOS, and it is considered more targeted to the dysregulation of the microcirculation in the setting of NO upregulation [3].

We report our institutional experience with the use of intravenous MB for refractory shock in patients admitted to the intensive care unit. In accordance with the current literature, we hypothesized that (1) at least 40% of critically ill patients would respond positively to MB administration and (2) that those who responded to MB would have a survival benefit [8]. Mortality in responders versus non-responders was assessed and a subgroup analysis in patients undergoing continuous renal replacement therapy (CRRT) was also performed.

## Methods

This study was approved by the institutional review board (Protocol #2020P000892), at Massachusetts General Hospital (Boston, MA, USA). The electronic medical records (Epic, Epic Systems Corporation, Verona, Wisconsin) of all patients ≥ 18 years of age admitted to an intensive care unit from October 2016 to May 2020 treated with MB for refractory vasodilatory shock were reviewed. Patients receiving extracorporeal membrane oxygenation (ECMO) or ventricular assist device (VAD) therapies were excluded. The dosing, timing and decision to administer MB was not protocolized and was at the discretion of the care team. Baseline demographics, indication for administration of MB, MB dose, method of administration (bolus with or without infusion), and duration of infusion were obtained. Additionally, MAP and vasopressor (norepinephrine, vasopressin, and epinephrine) infusion rates were recorded in the 1 hour prior to MB administration and at 1, 2, 4, 12 and 24 hours post-MB administration. Of note, this institution utilizes fixed dosing of vasopressors rather than weight-based and this was reflected in the data collection. Lastly, total fluid administered in the 24 hours post-MB administration, incidence of serotonin syndrome and glucose-6 phosphate dehydrogenase (G6PD) deficiency, use of CRRT, and survival to hospital discharge were also recorded.

MB responders were identified as those with a ≥ 10% increase in MAP within 1 to 2 hours after MB administration. Responders were compared to non-responders with respect to timing of MB administration, post-MB MAP and vasopressor requirements expressed in norepinephrine dose. Patients who did not have a reported MAP 2 hours post-MB were excluded from the analysis. We identified responders and non-responders to MB administration based on post-MB MAP and also assessed intensive care unit (ICU) mortality, ICU length of stay (LOS), hospital mortality, and hospital LOS.

### Statistics

Statistical calculations were performed in Microsoft Excel® (Redmond, WA, USA). Where appropriate, the comparison of averages (in responders and non-responders) and standard deviations of demographics, MB dose, MAP, and reductions in vasopressor dosing were performed via Chi squared, Fisher’s exact test, or two-tailed t-test with a p-value less than 0.05 considered as statistically significant. After using the F-test to assess for differences in variance, a two tailed t-test was used to compare sequential organ failure assessment (SOFA) scores among MB responders versus non-responders. A power analysis was waived given that this was a retrospective review that included all possible patients receiving MB.

## Results

**Fig. 1:**
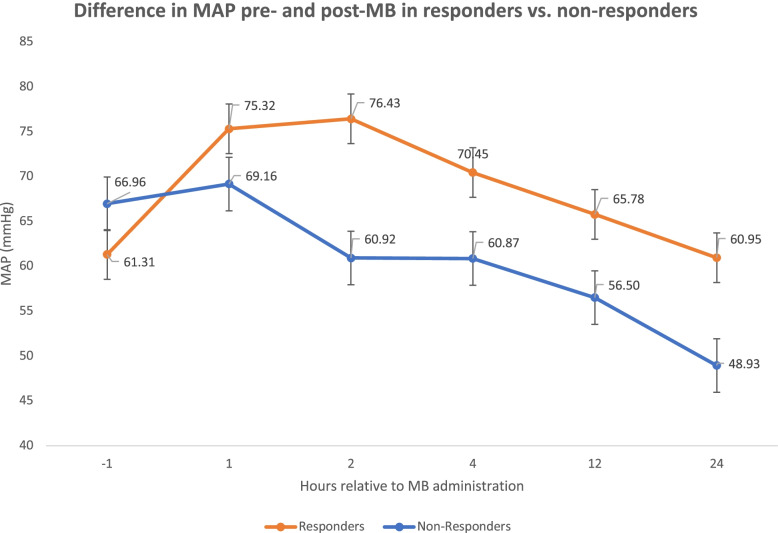
Difference in MAP pre- and post-MB in responders vs. non-responders

**Fig. 2:**
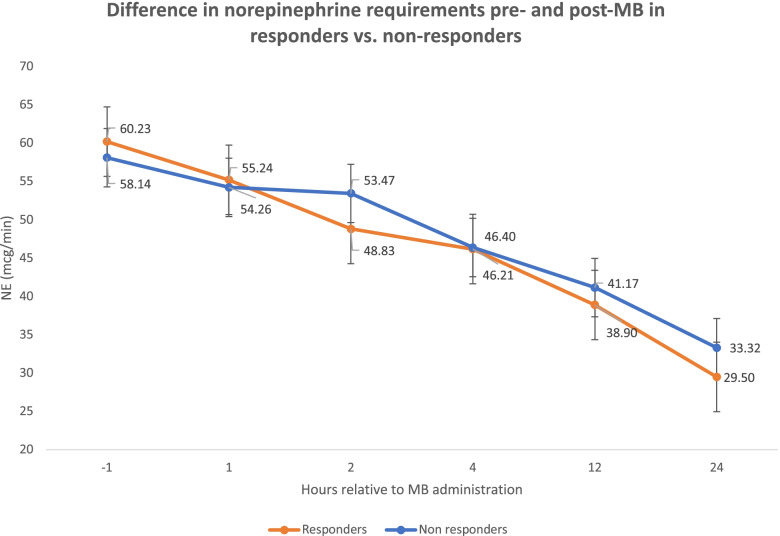
Difference in NE requirements pre- and post-MB in responders vs. non-responders

Between October 2016 to May 2020, we identified 306 ICU patients that received MB. After excluding patients receiving either ECMO or VAD therapies (*n*=70), 236 patients remained for analysis. A further 13 patients were excluded from any analysis comparing MB responders vs. non-responders due to incomplete data. Of the 223 patients included in the final MB cohort comparison analysis, 88 (39.5%) had a ≥10% increase in MAP in the first 1-2 hours post-MB administration and were considered MB responders whereas the remaining 135 (60.5%) patients were considered MB non-responders (Figs. 1 and 2). There was no significant difference in gender, age, weight, SOFA score, CRRT requirement, or norepinephrine dose 1 hour prior to administration of MB between groups. There was also no significant difference in administration of MB: bolus dose, use of infusion, infusion rate or duration between the responders and non-responders (Table 1). There was a statistically significant difference in volume resuscitation (within 24 hours after MB administration) between responders and non-responders to MB (3.6 ± 3.2L in responders vs. 2.5 ± 3.1L in non-responders) (*p*=0.01). The average MAP prior to MB administration was significantly lower in responders (61.3 ± 9.2 mmHg) compared to non-responders (67 ± 9.3) (*p* < 0.001). Following MB administration, the average MAP was higher in responders (76.4 ± 11.1) compared to non-responders (61 ± 15.5) (*p*<0.001).

**Table 1:** Responders versus non-responders^A^^,B^

	**Responders, *n*=88 (39.5%)**	**Non-Responders, *n*=135 (60.5%)**	***p*-value**
**Male gender (%)**	60 (68.2%)	95 (70.4%)	0.73
**Age (years)**	63 (13.4)	62 (15.5)	0.55
**Weight (kg)**	96.2 (24.3)	90.4 (25.1)	0.09
**MB bolus only (%)**	44 (50%)	85 (63.0%)	0.056
**MB bolus and infusion (%)**	39 (44.3%)	45 (33.3%)	0.098
**MB bolus dose (mg/kg)**	1.68 (0.49)	1.71 (0.43)	0.62
**> 1 MB bolus administered (%)**	14 (15.9%)	24 (17.8%)	0.72
**MB infusion rate (mg/kg/hr)**	0.48 (0.21)	0.45 (0.21)	0.54
**MB infusion duration (hours)**	11.8 (9.5)	16.7 (13.4)	0.06
**MAP 1 hour prior to MB (mmHg)**	61.3 (9.2)	67 (9.3)	**<0.001**
**MAP 2 hours after MB (mmHg)**	76.4 (11.1)	61 (15.5)	**<0.001**
**NE infusion rate 1 hour after MB (mcg/min)**	55 (39.1)	54 (42)	0.86
**NE infusion rate 2 hours after MB (mcg/min)**	49 (30.5)	53 (44.1)	0.41
**VP infusion rate 2 hours after MB (units/min)**	0.06 (0.03)	0.05 (0.02)	0.11
**EPI infusion rate 2 hours after MB (mcg/min)**	6.1 (3.9)	6.9 (5.8)	0.36
**Lactate 2 hours after MB (mmol/L)**	10.3 (6.1)	9.0 (5.2)	0.48
**SOFA Score**	13 (2.96)	13 (2.72)	0.31
**Survival to hospital discharge (%)**	19 (21.6%)	20 (14.8%)	0.19
**CRRT (%)**	57 (64.8%)	91 (67.4%)	0.68
**IV fluids administered in first 24 hours (mL)**	3645 (3221)	2548 (3143)	**0.01**

^A^ 13 patients did not have sufficient data to be included in the responders vs. non-responders group

^B^ Data reported as mean (+/- standard deviation) unless otherwise denoted with the percentage symbol

*Abbreviations*: *CRRT* Continuous renal replacement therapy, *IV* Intravenous, *MB* Methylene blue, *NE* Norepinephrine, *VP* Vasopressin, *EPI* Epinephrine, *SOFA* Sequential organ failure assessment

Responders were less likely to have chronic kidney disease (CKD) than those who were non-responders (21.6% vs 35.6%, *p*=0.03), but there were no differences in gender (*p*=0.53) or SOFA scores (*p*=0.15). There were no differences in the other investigated comorbidities between responders and non-responders including diabetes, atherosclerotic cardiovascular disease, chronic obstructive pulmonary disease, hypertension, hyperlipidemia, and chronic liver disease. There was one case of serotonin syndrome in the responder group noted after MB administration; no cases of hemolysis due to undiagnosed G6PD deficiency were reported in either group.

### Survival to discharge

A total of 88 (39.5%) patients had a ≥ 10% increase in MAP in the first 1-2 hours post-MB administration and considered MB responders, and this MAP increase was not associated with a statistically significant change in norepinephrine requirements when compared to non-responders (49 ± 30.5 mcg/min) vs. (53 ± 44.1 mcg/min) (*p*=0.41). Additionally, there was no statistically significant difference in vasopressin requirements between responders (0.06 ± 0.03 units/min) and non-responders (0.05 ± 0.02 unit/min) (*p*=0.11) or epinephrine requirements between responders (6.1 ± 3.9 mcg/min) and non-responders (6.9 ± 5.8 mcg/min) (*p*=0.36). The institutional culture is to titrate norepinephrine for MAP goals and remain on a fixed dose of vasopressin and epinephrine. There was a trend in improved survival to hospital discharge in the MB responder group compared to the non-responder group, but this result was not statistically significant (21.6% vs 14.8%, *p*=0.19).

### Additional results

Bolus doses of MB were 1.68 ± 0.5 mg/kg in responders vs 1.71 ± 0.4 mg/kg in non-responders (*p*=0.62). Responders tended to have an improvement in norepinephrine requirements within 1 hour after MB administration. Patients were separated into groups based on the indication for MB as documented and defined by the physician team (Table 2) into vasodilatory shock, 171 (72.5%), post-cardiotomy shock, 40 (16.9%), and other etiologies, 25 (10.6%). There were no statistically significant differences in survival based on these groupings. There was no difference in lactate level (mmol/L) 1 hour prior to MB administration between responders (8.7 ± 5.1) and non-responders (8.2 ± 5.4) (*p*=0.46). There was also no difference in lactate level 2 hours after MB administration between responders (10.3 ± 6.1) and non-responders (9.0 ± 5.2) (*p*=0.48).

**Table 2:** Grouping by shock etiology^A^

	**Overall n (%)**	**Responders, n (%)**	**Non-responders, n (%)**	***p*-value**
**Vasodilatory Shock**				
Total	171 (72.5)	63 (36.8)	95 (55.6)	
Survival ICU	13 (17.6)	4 (6.3)	12 (12.6)	0.242
**Post-cardiotomy Shock**				
Total	40 (16.9)	18 (45.0)	22 (55.0)	
Survival ICU	27 (67.5)	12 (66.7)	15 (68.2)	0.919
**Other Etiologies**				
Cardiogenic shock	5 (20)			
Mixed cardiogenic	12 (48.0)			
Mixed hemorrhagic	5 (20)			
**Allergic**	1 (4)			
Methemoglobinemia	1 (4)			
Non-cardiac surgical vasoplegia	1 (4)			
Total	25 (10.6)	7 (28.0)	18 (72.0)	
Survival ICU	2 (8.0)	2 (28.6)	0 (0)	N/A

^A^ Data presented as n (%) unless otherwise stated

*Abbreviations*: *ICU* Intensive care unit

In comparing hospital survivors (*n*=39, 16.5%) and non-survivors (*n*=197, 83.5%), there was no difference in gender, age, weight, or ICU LOS. Additionally, there was no significant difference in administration of MB in terms of dose of bolus, use of infusion, infusion rate or duration (Table 3). Hospital survivors were less likely to require CRRT (*p*<0.0001), had lower SOFA scores (*p*=0.0007), and had a longer hospital length of stay (*p*=0.004) compared to non-survivors (Table 2). Survivors had a significant increase in MAP compared to non-survivors at 2 hours (*p*=0.03), 4 hours (*p*=0.0001), and 12 hours (*p*=0.0001) post-MB administration. Concurrently, in survivors there was a significant decrease in norepinephrine requirements at 1 hour (*p*=0.0003) and 2 hours (*p*<0.0001) post-MB administration (Table 2).

**Table 3:** Hospital survivors versus non-survivors^A^^,B^

	**Survivors, *n*=39 (16.5%)**	**Non-survivors, *n*=197 (83.5%)**	***p*-value**
**Male gender (%)**	30 (76.9%)	154 (78.2%)	0.863
**Age (years)**	63 (14.9)	62 (15.4)	0.88
**Weight (kg)**	88.6 (14.2)	92.8 (26.3)	0.33
**MB bolus only**	25 (64.1%)	115 (58.4%)	0.51
**MB bolus and infusion**	14 (39.5%)	72 (36.6%)	0.94
**MB bolus dose (mg/kg)**	1.7 (0.42)	1.7 (0.45)	0.97
**MB drip rate (mg/kg/hr)**	0.39 (0.22)	0.48 (0.21)	0.15
**MB drip duration (hours)**	13.9 (10.9)	14.3 (12.3)	0.91
**NE at 1 hours after MB (mcg/min)**	34.5 (33)	61 (42)	**0.0003**
**NE at 2 hours after MB (mcg/min)**	27.7 (18.2)	57 (40.5)	**<0.0001**
**>1 MB bolus administered (%)**	7 (17.9%)	32 (16.2%)	0.79
**CRRT (%)**	16 (41%)	137 (69.5%)	**<0.0001**
**IV fluids administered in first 24 hours (mL)**	2352 (3096)	2951 (3186)	0.28
**ICU LOS (days)**	13.9 (21.9)	11.5 (21.8)	0.53
**Hospital LOS (days)**	28.8 (28.5)	15.3 (25.9)	**0.004**
**SOFA Score**	11.69 (2.2)	13.35 (2.9)	**0.0007**

^A^ Total number of patients who received MB and were assessed was 236

^B^ Data reported as mean (+/- standard deviation) unless otherwise stated with percentage symbols

*Abbreviations*: *CRRT* Continuous renal replacement therapy, *ICU* Intensive care unit, *IV* Intravenous, *LOS* Length of stay, *MB* Methylene blue, *NEE* Norepinephrine equivalents, *SOFA* Sequential organ failure assessment

Patients undergoing CRRT were additionally analyzed and identified as responders based on a norepinephrine dose requirement decrease of ≥10% after two hours. The 70 patients requiring CRRT were analyzed as a subgroup; 33 were found to be MB responders and 37 were non-responders. There were 12 survivors in the responder group (63.6% hospital mortality) and 4 survivors in the non-responder group (89.2% hospital mortality). Within the CRRT subgroup, hospital mortality was significantly lower in the responder group (*p* = 0.0111) despite a lack of difference in SOFA score (12.96 ± 2.64 vs 14 ± 2.34, *p* = 0.16) or gender (*p* = 0.48).

## Discussion

There are currently limited pharmacologic therapies for patients presenting with vasoplegia. In this retrospective single institutional study, 233 patients received MB in the setting of shock (at least 72.5% of which were vasodilatory), of which 88 (39.5%) had a ≥ 10% increase in MAP within two hours of administration. Although not statistically significant, there was a trend toward improved survival to hospital discharge in patients with a response to MB. The dosing of MB may also play a role in identifying responders. Although the results were not statistically significant, MB responders were more likely to receive bolus dosing plus an infusion (44.3%) when compared to non-responders (33.3%). There was not a fixed protocol for dosing administration in this retrospective study and the pharmacokinetics of the drug may favor bolus and infusion dosing over bolus only dosing; the average duration of infusion for MB responders was 11.8 ± 9.5 hours.

The use of MB for vasodilatory shock has been described in the literature over the last thirty years in the setting of sepsis, post-cardiopulmonary bypass vasoplegia, anaphylaxis, liver failure, and drug-induced shock [1, 9, 10]. Although studies have shown improvements in MAP and systemic vascular resistance from baseline after MB administration, a statistically significant improvement in survival has not been reliably shown [11–18]. A multicenter, randomized, double-blind, placebo-controlled trial of a nitric oxide synthase (NOS) inhibitor, 546C88, in septic shock found a higher likelihood of resolution of shock at 72 hours [19]. However, a subsequent larger trial of treatment with 546C88 in patients with septic shock was halted early due to a statistically significant increase in 28 day mortality due to cardiovascular causes [20]. Following this study, the further investigation of NOS inhibitors lost traction, however, a closer look at the findings suggests that some patients with very high cardiac outputs may have derived benefit. Furthermore, MB may target iNOS as opposed to 546C88 which is a non-specific NOS inhibitor [7].

Despite the lack of mortality benefit in prospective trials with NOS inhibitors, more recent retrospective studies have aimed to identify whether or not there is a subgroup of patients that may benefit from MB. Studies that have separated patients by defining an immediate clinical response to MB administration via MAP increase or vasopressor requirement decrease suggest that there may be a survival benefit in patients that are responders to MB [8, 21, 22]. In the study by Porizka, it was shown that MB responsiveness may associated with lesser degree of tissue hypoxia in critically ill patients [21]. In this study, there was no difference in degree of tissue hypoxia as defined by lactate in the responders and non-responders prior to MB administration or two hours after administration.

We found that 88 (39.5%) patients were responders to MB based on an increase in MAP of ≥ 10% at 2 hours following MB administration. The study by Mazzeffi et al found that 44% of patients with post-cardiotomy shock were responders to MB as defined by a 20% increase in MAP following MB administration [8]. The stark difference in survival comparing our study, with responders having a 78.4% mortality, to the Mazzeffi study, where responders had an 8.3% hospital mortality, is most likely attributed to the frequent use of MB in cases other than post cardiopulmonary bypass (CPB) vasoplegia (a circumstance in which a defined insult, CPB, is terminated as opposed to sepsis which is an ongoing driver for vasodilatory shock). It is challenging to design an adequately powered prospective study of MB in patients with vasodilatory shock because the etiology of vasodilation likely plays a major role in survival. Designing a trial around the premise of responders and non-responders is important as the literature suggests that responders to MB may have a trend towards decreased mortality [8, 21, 22]. Designing a study around responders to MB would potentially allow for prognostication of survival based on the response to MB.

Exploring this concept further should also include whether the duration of vasodilation prior to MB administration plays a role in identifying responders versus non-responders. A randomized controlled study in cardiac surgical patients at high risk of post-operative vasoplegia demonstrated that preoperative MB administration reduced the incidence and severity of vasoplegic syndrome in both the operative and postoperative time frames and reduced ICU and hospital length of stay [23, 24]. Mehaffey et al subsequently found that early (in the operating room) administration of MB was associated with improved survival and a reduction of the risk-adjusted rate of major adverse events compared to late (in the ICU) administration of MB in cardiac surgical patients [25]. There may be a role for identifying high risk patients for vasoplegia outside of the cardiac surgical population and studying early versus late MB administration. The use of MB in this study was at the discretion of the care team and not based on a protocol. Identifying “trigger” situations where patients may qualify for MB administration based on persistently low MAP in the setting of appropriate volume resuscitation may allow for better standardization and study of the potential benefit of MB for vasoplegia.

The results of the subgroup analysis of patients undergoing CRRT are intriguing. Patients who required CRRT had significantly higher SOFA scores (13.5 ± 2.5) than those patients who did not require CRRT (11.3 ± 2.0) (*p*<0.001). In the subgroup of patients supported by CRRT that were identified as MB responders, hospital mortality was lower (63.6%) compared to those who did not respond to MB (89.2%). This may be due to the reduced clearance of MB in patients requiring CRRT, effectively increasing the amount of drug the patients were exposed to which may also inform the dose of MB that is required to provide benefit. Tumlin et al demonstrated that patients with vasodilatory shock and acute kidney injury requiring renal replacement therapy had an improved MAP response and survival when treated with angiotensin II compared with placebo [26]. In another study, MB administration demonstrated a renoprotective effect with improved creatinine clearance and decreased excretion of tubular damage markers, which could partially explain the improved clinically outcome of MB responders [27]. It may be that responders within this CRRT subgroup to rescue agents (such as MB and angiotensin II) derived greater survival benefit. The need for renal replacement therapy in the setting of shock is associated with poor outcomes and if this finding is replicated in other cohorts, perhaps MB could play a special role in this patient population. A prospective study comparing the administration of methylene blue to angiotensin II in patients undergoing CRRT with vasoplegia is warranted to determine the relative efficacy of the two agents in this patient population.

Future areas of (ideally prospective) research must include answering the question of whether certain sub-populations are more likely to respond to MB such as those supported with ECMO, those suffering from post-cardiotomy shock, and those requiring CRRT [8, 22].

### Limitations

Limitations of this study include the retrospective nature, the single-institution experience, and the non-standardized approach to therapy. Additionally, the diagnosis of vasoplegia was made clinically with the etiology defined by the care team. There was not universal monitoring for cardiac index to delineate normal versus hyperdynamic cardiac physiology. There are no consensus guidelines or defined institutional practices regarding timing or clinical triggers for MB administration, therefore, the use of MB was physician dependent. The duration of vasoplegia and inotropic support prior to the administration of MB was not uniform and could theoretically play a role in the survival benefit among MB responders. However, the non-significant difference of SOFA score between MB responders and non-responders suggests a similar shock severity prior to treatment. Finally, the use of additional non-adrenergic treatments for vasoplegia including corticosteroids, hydroxocobalamin, and angiotensin-II were not recorded in this dataset, though hydroxocobalamin is not used in our institution outside of the cardiac surgical realm.

The dosing and administration of MB was not uniform and it may be that a higher dose of MB (2-3 mg/kg) with a higher infusion rate (0.5 mg/kg/hr) could have had a more pronounced hemodynamic effect [5]. Additionally, the amount of fluid resuscitation could have confounded the results as the non-responders received significantly less resuscitation than the responders. The specific resuscitation fluid was also not standardized or documented in this dataset and patients in either group may have received a combination of crystalloid, colloid, and/or blood products at the discretion of the care team.

Additionally, a formal power analysis could not be performed given that this was a retrospective study. The subgroup analysis of CRRT patients is suggestive of a population that may have particular benefit from MB, however, the sample size is small and further research is required to verify this finding.

## Conclusion

In patients with refractory shock receiving MB, identifying patients as responders and non-responders within two hours of administration based on a MAP increase ≥10% shows a non-statistically significant trend toward improved outcomes in responders. Further research is necessary to identify patients who may receive the most benefit from MB therapy.

## Data Availability

The datasets used and/or analyzed during the current study are available from the corresponding author on reasonable request.

## Abbreviations

MB: Methylene blue
MAP: Mean arterial pressure
CRRT: Continuous renal replacement therapy
NO: Nitric oxide
sGC: Soluble guanylate cyclase
cGMP: Cyclic guanosine monophosphate production
CI: Cardiac Index
eNOS: Endothelial nitric oxide synthase
iNOS: Inducible nitric oxide synthase
TNF-α: Tumor necrosis factor-α
ECMO: Extracorporeal membrane oxygenation
VAD: Ventricular assist device
G6PD: Glucose-g-phosphate deyhydrogenase
ICU: Intensive care unit
LOS: Length of stay
SOFA: Sequential organ failure assessment
CKD: Chronic kidney disease
NOS: Nitric oxide synthase
CPB: Cardiopulmonary bypass
